# Adverse Effects of Virtual and Augmented Reality Interventions in Psychiatry: Systematic Review

**DOI:** 10.2196/43240

**Published:** 2023-05-05

**Authors:** Robert M Lundin, Yuhern Yeap, David B Menkes

**Affiliations:** 1 Change to Improve Mental Health Mental Health Drugs and Alcohol Services Barwon Health Geelong Australia; 2 Institute for Mental and Physical Health and Clinical Translation Deakin University Geelong Australia; 3 Waikato Clinical Campus University of Auckland Hamilton New Zealand; 4 Mental Health and Addictions Waikato District Health Board Hamilton New Zealand

**Keywords:** virtual reality, augmented reality, mental health, side effects, adverse events, hardware, VR, software, AR, cybersickness, reporting standards

## Abstract

**Background:**

Virtual reality (VR) and augmented reality (AR) are emerging treatment modalities in psychiatry, which are capable of producing clinical outcomes broadly comparable to those achieved with standard psychotherapies.

**Objective:**

Because the side effect profile associated with the clinical use of VR and AR remains largely unknown, we systematically reviewed available evidence of their adverse effects.

**Methods:**

A systematic review was conducted in accordance with the PRISMA (Preferred Reporting Items for Systematic Reviews and Meta-Analyses) framework across 3 mental health databases (PubMed, PsycINFO, and Embase) to identify VR and AR interventions targeting mental health diagnoses.

**Results:**

Of 73 studies meeting the inclusion criteria, 7 reported worsening clinical symptoms or an increased fall risk. Another 21 studies reported “no adverse effects” but failed to identify obvious adverse effects, mainly cybersickness, documented in their results. More concerningly, 45 of the 73 studies made no mention of adverse effects whatsoever.

**Conclusions:**

An appropriate screening tool would help ensure that VR adverse effects are correctly identified and reported.

## Introduction

### Overview

Substantial unmet need for treatment for mental disorders arises from problems of access, efficacy, and tolerability of conventional treatments [1,2]. These limitations have prompted the development and evaluation of novel interventions, including those based on virtual reality (VR) and augmented reality (AR) [3,4]. Recent systematic reviews and meta-analyses have shown that VR and AR can be usefully applied to the treatment of various psychological disorders [5-7], mainly using versions of cognitive behavioral therapy (CBT) and the specific CBT technique of exposure therapy (ET) [8]. Despite the enhanced accessibility and scalability of digital interventions, individual VR studies are often criticized for their limited quality and sample size [9].

Despite its therapeutic promise, the stance of VR-based psychotherapy cannot be established without a realistic appraisal of its benefits and harms. Unfortunately, little evidence is available regarding VR side effects experienced by mental health cohorts, a deficit shared with psychotherapies in general [10-12]; both lack comprehensive identification of adverse effects, including symptom deterioration [13]. There is an urgent need to improve detection, reporting, and evaluation of adverse effects of psychotherapies, including VR [14].

### Adverse Effects in VR and AR Clinical Trials

We define adverse effects as those perceived as unpleasant or harmful by the patient, clinician, or family, including symptomatic deterioration [11], addiction, or delusions manifesting in relation to VR use [10]. Previous studies refer to VR-induced symptoms and effects involving nausea, dizziness, disorientation, postural instability, and fatigue. These unpleasant physical symptoms experienced are often referred to as “cybersickness,” which is found to be negatively correlated with the subjective experience of “presence” in the virtual environment [15]. VR interventions can also induce dissociative symptoms [16], including perceived disconnection from the self (depersonalization) and the environment (derealization).

The full mechanism for the above experiences is not known, but they are thought to be triggered by the discontinuity between digital and objective reality. While the frequency and severity of these are being reduced by technological improvements in hardware and software, as well as a reduction in the time spent in VR, the effects are often subjective experiences that cannot be mitigated in some individuals and vary greatly based on the hardware and software used [17]. Relevant hypotheses primarily involve the mismatch between the 2 sensory systems involved in motion detection. If a user is standing still, no motion is detected by the vestibular system; however, when using a VR device, there is a direct contradiction owing to the motion being observed by the visual system [18]. Measuring and addressing such adverse effects is important because they predict poorer treatment outcomes and increased nausea [19-21]. ET, whether in vivo or delivered via VR, can transiently intensify distress [22] while improving outcomes overall [23].

This has led to the development of rating scales for cybersickness, including the Simulator Sickness Questionnaire (SSQ), Visual Analogue Scale, the Fast Motion Sickness Scale, and Virtual Reality Sickness Questionnaire [24-26]. It has also led to the repurposing of previously reported scales such as Subjective Units of Distress Scale [27] and, more recently, the development of the Virtual Reality Neuroscience Questionnaire [28], which additionally measures user experience with the software, allowing direct comparisons between different interventions. There are also tools specifically scoring the degree of “presence” such as the Igroup Presence Questionnaire [22]. This is important because the intervention's ability to influence the emotional state, fundamental to therapeutic effects, is related to the sense of presence and can be disrupted by adverse effects interrupting the simulation [25].

Because VR studies often lack a standardized research design, adverse effect reporting is inconsistent and needs to be improved to reckon VR’s appropriate place in mental health [6,29]. Recommendations for the design of VR clinical trials, developed by an international consortium, address the safety and tolerability of equipment, headset, and intervention. Specific recommendations have highlighted the importance of assessing the psychological and emotional experiences of each participant [30]. Considering the prevalence of physical symptoms reported in the wider VR literature, it is concerning that mental health applications rarely report physical or psychological adverse effects [10,31,32], such as reduced cognitive performance [33,34], physical or eye fatigue [35], and cybersickness [36]. This review examines adverse effects reported in trials of VR and AR in mental health.

## Methods

### Identification and Selection of Studies

**Figure 1:**
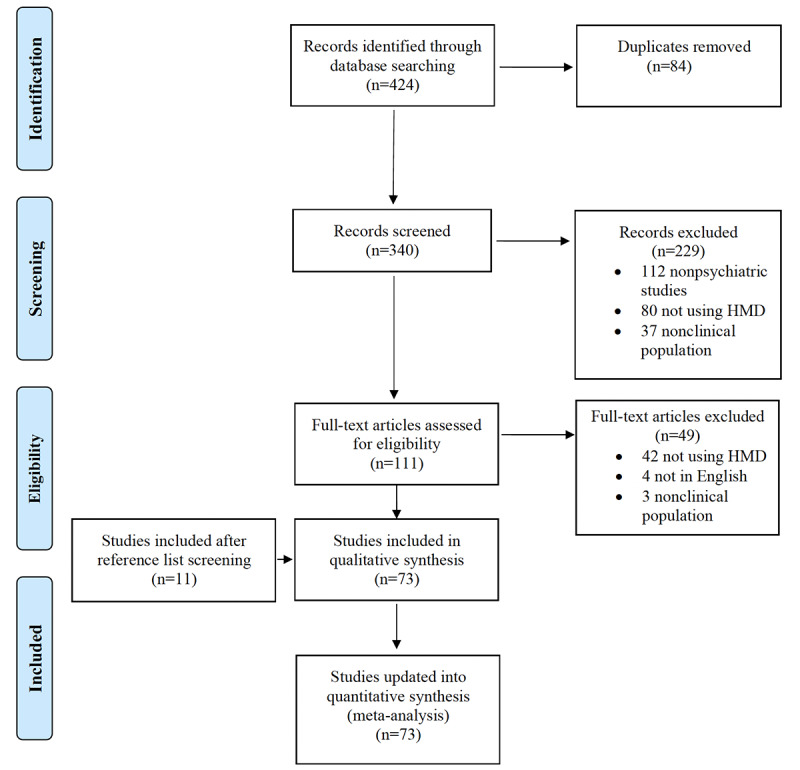
PRISMA (Preferred Reporting Items for Systematic Reviews and Meta-Analyses) study selection flowchart. HMD: head-mounted display.

A systematic search of 3 databases (PsycINFO, PubMed, and Embase) concluded on September 17, 2022. These databases were selected as the 3 largest repositories for mental health studies. The search included the terms “virtual reality” or “augmented reality” or “computer-assisted” in combination with a range of mental health disorders to capture a breadth of relevant conditions including “mental health” or “psychiatry” and “treatment” or “therapy” or “intervention” or “psychotherapy” or “attention deficit hyperactivity disorder” or “dementia” or “cognitive impairment” or “depression” or “mood disorder” or “schizophrenia” or “psychosis” or “psychotic” or “phobia” or “anxiety” or “bipolar” or “PTSD” or “post-traumatic stress disorder” or “alcohol” or “substance” or “anorexia” or “bulimia“ or “eating disorder” or “psychiatric” or “mental illness” or “mental health.” The reference lists of studies included for full-text review were used to identify additional articles not captured by the initial search process. The literature search was conducted in accordance with PRISMA (Preferred Reporting Items of Systematic reviews and Meta-Analyses) guidelines as depicted in Figure 1. Duplicate studies were identified and removed; the remaining studies were screened by 2 authors (YY and RL).

### Eligibility Criteria

Screened papers were included for analysis if they (1) used a VR- or AR-based intervention administered via a head-mounted display (HMD) and (2) addressed a specific Diagnostic and Statistical Manual of Mental Disorders or International Classification of Diseases mental health disorder. The authors decided to focus on the use of HMDs because the term “virtual reality” is often used to reference nonimmersive alternatives such as computer screens or Cave Automatic Virtual Environments. If both conditions were not met, the paper was excluded. Final decisions on inclusion were reached by consensus, with papers grouped on the basis of disorder and data type. Selected papers were examined for information on the type of mixed reality used, psychological intervention implemented and target disorder, the number of patients in the data set, reported side effects, and any measure of presence or immersion.

## Results

### Study Selection

The systematic search of databases yielded 424 hits (Figure 1), with 84 duplicates. The examination of abstracts led to the exclusion of 85 articles, leaving 111 for full-text examination. In total, 42 studies were excluded for not using an HMD, 4 had no available translation, and 3 were based on nonclinical populations. Screening of reference lists yielded another 2 admissible reports. In total, 73 studies used VR or AR to treat a psychiatric diagnosis and were selected for analysis.

### Study Characteristics

Of the 73 included studies, 67 used VR, 3 used AR, 2 used both VR and AR, and 1 used mixed reality. The main psychological intervention used was ET (n=52, 71%), followed by CBT without ET techniques (n=12, 16%). Non–CBT-based interventions (n=9, 12%) included embodiment illusion (n=2) and provision of psychoeducation (n=1), relaxation (n=1), cognitive rehabilitation (n=1), physical and cognitive training (n=1), body swapping (n=1), interreality (n=1), and aggression prevention therapy (n=1). A variety of specific problems were targeted by these interventions, which are outlined in Table 1.

Of 73 studies included, only 22 measured levels of immersion or presence. The most popular rating tool was the Igroup Presence Questionnaire (n=6). Other tools included the SSQ (n=3), Presence and Reality Judgment questions (n=3), and an embodiment questionnaire (n=3) where 3 other measures were used once each (n=1). The remaining studies used unknown or subjective descriptions of presence or immersion (n=7).

**Table 1:** Mental disorders targeted by virtual reality and augmented reality in the included studies.

Mental disorder	Studies, n
Phobias	30
Posttraumatic stress disorder	17
Anxiety	10
Psychosis	7
Eating disorders	6
Stress	3
Cognitive impairment	1
Suicidal ideation	1
Aggression	1
Addiction	1
Depression	1

### Reporting of Adverse Effects

Most studies (45/73, 62%) made no reference to adverse or side effects. Of the remaining 28, a total of 21 studies reported no adverse effects, but in 5 of them, adverse effects were apparent but not identified as such (Table 2).

Seven studies reported adverse effects but provided limited information (Table 3). Overall, the use of a tool that measured presence, immersion, or cybersickness was more common in the studies that commented on adverse effects (12/29, 27%) than in those that did not comment on adverse effects (12/44, 31%).

Data on dropouts from treatment groups were reported by 49 of 73 studies, but only 6 reported relevant reasons, including failure to arouse anxiety (n=2), high anxiety (n=2), anxiety along with a lack of engagement (n=1), finding the VR equipment “too distracting,“ distress from previous physical or sexual abuse, and avatars being “too unrealistic” (n=1).

**Table 2:** Adverse effects detected in studies reporting “no adverse effects.”

Study (year)	Participants in the exposure group, n	Adverse effect described
Pot-Kolder et al (2018) [37]	58	Dropouts due to cybersickness (n=1) and uncomfortable devices (n=2)
Pericot-Valverde et al (2015) [38]	41	Increased cigarette craving (n=4)
Nason et al (2019) [39]	7	Mild motion sickness (n=1)
Gujjar et al (2018) [40]	5	Cybersickness (n=4)
Veling et al (2021) [41]	50	Dropouts due to cybersickness (n=2)

**Table 3:** Studies reporting adverse effects.

Study (year)	Participants in the exposure group, n	Adverse effect described
Maltby et al (2002) [42]	20	Dropout due to cybersickness (n=1)
Reger et al (2016) [43]	52	Worsened symptoms of posttraumatic stress disorder (n=1)
Botella et al (2016) [44]	32	An unspecified number of participants reported tiredness, dizziness, or back pain, prompting a change in hardware
Krijn et al (2007) [45]	29	Dropout due to simulator sickness (n=1)
Levy et al (2016) [46]	9	“Walked carelessly” after therapy, judged to be at risk of falling (n=1)
Gujjar et al (2019) [47]	15	Mean increase in cybersickness rating post virtual reality
Kim et al (2020) [48]	32	Anxiety scores increased halfway through treatment (session 4) and then decreased

## Discussion

This review highlights significant gaps in reporting the adverse effects of VR interventions in mental health. In a majority of included articles, authors made no mention whatsoever of possible adverse effects associated with technology. The identified studies specifically mention cybersickness, worsening of symptoms (posttraumatic stress disorder, anxiety, and cravings), tiredness, dizziness, back pain, and carelessness. Also concerning was the fact that even studies that mentioned possible adverse effects still did not recognize and report them as such. Available evidence indicates that adverse effects associated with VR are likely to be common, but the identified lack of good data makes it difficult to estimate rates with any confidence. Some studies also specifically focus on measures of immersion or presence or only report serious adverse events that would involve significant harm or death. While this is a valid approach for traditional clinical trials with large cohorts focused on medications with serious side effects, it is unlikely to be a sufficient approach for VR interventions in mental health. What is clear from these limited results is that use of VR and AR in mental health studies has adverse effects that include traditional vestibular-related side effects, physical experiences, and psychological impacts, which will all need to be considered.

Although recent attempts have been made to formulate a research framework for these interventions, these have not addressed the detection and reporting of adverse reactions. It is, therefore, important that robust protocols are developed to rectify this shortcoming [30,49]. This review highlights that fewer than half of all VR mental health studies report adverse effects. This points to a significant problem with reporting standards in these studies, which need to be addressed as the initial concern. This is particularly important because adverse effects can significantly influence the subject's emotional state and, therefore, the therapeutic effect of the intervention [16].

It is puzzling that many studies would use simple subjective measures of presence despite the many validated tools available. It has been established that the use of personal digital avatars, and especially seeing your own body in the virtual space, has a strong effect on presence [50]. Such factors are rarely discussed in mental health studies, which is an important omission as it would be particularly key in studies on eating disorders where participants are occasionally asked to assess body sizes.

Future studies will need to consider the range of physical and psychological adverse effects associated with AR and VR interventions and use screening tools that include physical symptoms such as cybersickness and VR-induced symptoms and effects, symptoms of dissociation, negative emotional responses such as increased rumination or thoughts of self-harm, and wider impacts on functioning. Although this would require the use of several tools, an ideal focus for future work would be to use a questionnaire covering all sections to standardize reporting of VR and AR interventions in mental health. This would include a move from older measures such as the SSQ tool to more dimensional assessments using the Virtual Reality Neuroscience Questionnaire tool, which provides grading and a broader approach to assess interventions. This could include the incorporation of established tools such as the Clinician-Administered Dissociative States Scale for dissociation and the Experience of Therapy Questionnaire [51].

As with the evaluation of any health care intervention, a benefit-risk analysis should be standard, where the dimension of risk includes the safety profile identified from the range and frequency of adverse effects. It is essential that all adverse effects, especially those that lead to dropouts or clinical worsening, need to be identified and reported. It is of concern that some studies specifically excluded participants experiencing cybersickness as the adverse events reported will not be representative of the general population. Thorough reporting would allow future systematic reviews to include meta-analyses of outcomes, including adverse effects, and improve our understanding of benefits, harm, and the appropriate place of VR and AR in psychiatric treatment.

This systematic review has multiple limitations. The study design focused specifically on HMDs and does not incorporate the wider (and generally older) literature regarding computer-generated environments projected onto screens. It also focused on diagnosed psychiatric disorders, and the results cannot be generalized to wider health care and nonclinical populations. There was also significant heterogeneity in the definitions of VR and AR in the literature, to the extent that our otherwise detailed search may not have captured some studies. Only papers published in English were included in the review.

Despite promising developments in VR and AR across a range of mental disorders, there is a clear need for standardized detection and reporting of adverse effects associated with these interventions.

## Abbreviations

AR: augmented reality
CBT: cognitive behavioral therapy
ET: exposure therapy
HMD: head-mounted display
SSQ: Simulator Sickness Questionnaire
VR: virtual reality
